# Time in Bed Is Associated with Decreased Physical Activity and Higher BMI in Women Seeking Weight Loss Treatment

**DOI:** 10.5402/2012/320157

**Published:** 2012-11-08

**Authors:** Chantelle N. Hart, Joseph L. Fava, Leslee L. Subak, Katie Stone, Eric Vittinghoff, Kathryn E. Demos, Erin O'Brien, Alyssa Cairns, Rena R. Wing

**Affiliations:** ^1^Weight Control and Diabetes Research Center, The Miriam Hospital and Department of Psychiatry and Human Behavior, Alpert Medical School of Brown University, 196 Richmond Street, Providence, RI 02903, USA; ^2^Department of Urology, University of California, San Francisco and UCSF Women's Health Clinic Research Center, 1635 Divisadero Street, Suite 600, San Francisco, CA 94143-1793, USA; ^3^San Francisco Coordinating Center, University of California, San Francisco and California Pacific Medical Center Research Institute, Suite 5700, 185 Berry Street, San Francisco, CA 94107, USA; ^4^Division of Biostatistics, Department of Epidemiology and Biostatistics, University of California, San Francisco, 185 Berry Street, Lobby 5, Suite 5700, San Francisco, CA 94107, USA

## Abstract

Short sleep duration is associated with obesity risk. Despite calls to incorporate strategies to enhance sleep within the context of behavioral weight loss (BWL) treatment, little is known regarding the association between sleep and body mass index (BMI) among individuals presenting for BWL. Moreover, most research has focused on eating pathways linking sleep and BMI and has not explored how sleep may impact engagement in physical activity. The purpose of the present study was to determine whether, in a sample of women seeking treatment for weight loss, there was an association between reported time in bed (TIB), higher BMI, lower physical activity, and less favorable dietary composition. Prior to randomization, 318 women completed measures of TIB, eating, and activity; weight and height were measured. Findings demonstrated that report of “6 hours or less” TIB/night was associated with higher BMI and lower reported physical activity compared to that of the referent (>7 to ≤8 hours/night). It was not associated with the number of reported calories consumed each day or with the percent of calories consumed from fat, carbohydrates, or protein. Better understanding of the role of sleep within the context of BWL treatment in women seems warranted.

## 1. Introduction

Thirty-six percent of adults in this country are obese [1]. Notably a comparable percentage (35–37%) also report sleeping less than seven hours per night [2, 3]. A number of epidemiological studies have demonstrated a significant association between short sleep duration and obesity status in both children and adults with a meta-analysis showing that for every hour less that adults sleep per night, there is a 0.35 kg/m^2^ increase in BMI [4]. One recent study found that the association between reported short sleep duration and obesity risk appears to be more pronounced in women [5]. Thus findings point to the need to better understand whether sleep duration may be an important factor to target within behavioral weight loss (BWL) treatment, particularly in women.

A number of studies have explored how eating pathways may link short sleep with obesity risk. Mounting evidence suggests that sleep may influence weight status through its effect on the neuroendocrine control of appetite and food intake [6–8]. Specifically, the hormones leptin and ghrelin, which are associated with the regulation of hunger and appetite, are influenced by sleep [7–9]. Studies that have assessed the influence of sleep on reported or observed food intake, however, have demonstrated mixed findings. Some found no effect of sleep length on food intake in either naturalistic [10] or experimental [11, 12] settings, while others have demonstrated that short sleep is associated with reported increases in snack food intake [13] and observed increases in ad libitum food intake (compared to rested conditions) in experimental settings [5, 14]. These studies have primarily focused on healthy weight adults. Thus generalizability of findings to obese, treatment-seeking populations may be limited. 

Less research has focused on activity pathways linking sleep and BMI, yet, it is quite plausible that individuals who carry a sleep debt (i.e., chronically not getting enough sleep) will have greater homeostatic pressure for sleep than for engagement in physical activity [15]. However, observational and experimental findings on the association between sleep duration and physical activity have been mixed [5, 11, 12, 16, 17]. Given that few studies have been conducted thus far it is important to further understand how engagement in physical activity may be affected by sleep duration, particularly in individuals who are overweight or obese and who would be asked to increase physical activity within the context of BWL treatment. 

Despite their limitations and mixed results, findings from studies described above have been used to argue that sleep duration should be enhanced to promote weight loss [18, 19]. However, the association between sleep and obesity status has rarely been assessed within BWL treatment-seeking populations. Prior to drawing conclusions regarding the utility of enhancing sleep for weight loss, it is important to first establish whether short sleep is associated with obesity risk in a treatment-seeking population. The purpose of the present study was to assess the association between time in bed (TIB; i.e., the time between getting into bed and trying to fall asleep and waking the next day) and obesity risk in a sample of women presenting for BWL treatment. A convenience sample of women who participated in a randomized controlled trial that assessed the effect of weight loss on urinary incontinence (Program to Reduce Incontinence by Diet and Exercise; PRIDE) [20] was used. This population was chosen given the potential increased risk for higher BMI in short sleeping women [21] as well as the need to further characterize the association between sleep and obesity risk in a treatment-seeking sample. It was hypothesized that shorter self-reported TIB would be associated with higher BMI, lower physical activity, and less favorable dietary composition.

## 2. Methods

### 2.1. Participants

Participants in a previously published clinical weight loss trial (PRIDE) [20] were included in the present analyses. All women in the trial were at least 30 years of age, were overweight or obese (BMI of 25–50 kg/m^2^), and reported at least 10 urinary incontinent episodes per week on a voiding diary. A total of 338 women were enrolled in PRIDE from July 2004 to April 2006. For the present paper, women with suspected shift work or circadian disturbance (*N* = 7; 2%), defined as self-reported initiation of sleep onset after 6:00 am or prior to 7:00 pm, were excluded from the study sample. Due to extreme values on the Block Food Frequency Questionnaire, we chose to trim the data of outliers, which resulted in excluding two (<1%) participants. To assess racial differences for sleep and BMI, participants who did not report being either Caucasian or African American/Black (*N* = 11; 3%) were also excluded. This resulted in 318 (94%) women being included in analyses. Participants included in the present study did not differ from those excluded on age, BMI, total weekly urinary incontinence episodes, physical activity, or any of the dietary composition variables assessed.

### 2.2. Procedures

Procedures for this randomized controlled trial have been described previously [20]. The study was approved by the Institutional Review Boards at all sites; written informed consent was obtained prior to participant enrollment. Briefly, demographic characteristics, medical, behavioral, incontinence histories, TIB, eating, and activity behaviors were ascertained using self-reported questionnaires completed at the baseline assessment. All measures were completed prior to participants being randomized into treatment condition. 

### 2.3. Measures

#### 2.3.1. Anthropometrics

Trained staff measured participants' weight and height with participants wearing street clothes and without shoes. Weight was measured to the nearest 0.5 kg on a calibrated digital scale (Tanita BWB 800) and height to the nearest centimeter using a calibrated, wall-mounted stadiometer. Weight and height data were used to calculate participant BMI (kg/m^2^).

#### 2.3.2. Demographics, Health, and Lifestyle Behaviors

Participants reported on a number of demographic, health, and lifestyle behaviors, including the following: age, race, education, relationship status, employment, reported alcohol consumption, and frequency of snoring/coughing loudly (i.e., as a marker of sleep disordered breathing). They also completed the Beck Depression Inventory to report on depressive symptoms.

#### 2.3.3. Urinary Incontinence

Participants completed a 7-day voiding diary, which was reviewed by trained research staff. This included documenting the time of each incontinence episode and type of episode: stress (involuntary loss of urine due to cough, sneeze, strain, or exercise), urge (loss of urine due to need/urge to void), or other. For analyses, women were classified as having predominantly stress, predominantly urge, or mixed incontinence. Voiding diaries are reliable and valid measures of urinary incontinence episodes [22, 23]. For the present study, total nightly and total weekly incontinence episodes as well as type (stress, urge, or mixed) were of primary interest.

#### 2.3.4. Dietary Intake

The Block Food Frequency Questionnaire (Block FFQ) is a widely used reliable and valid measure of food intake [24]. It consists of 110 food items and is designed to estimate typical food intake across food groups. For the present paper, variables of interest included kilocalories (kcal), percent kcal from fat, percent kcal from protein, percent kcal from carbohydrates, and percent kcal from sweets.

#### 2.3.5. Physical Activity

The Paffenbarger Physical Activity Questionnaire (PPAQ) is a widely used self-report measure of lifestyle physical activity [25, 26]. Participants are asked to report on stairs climbed, walking, and engagement in sports and recreation during the previous week (or if unusual, on a typical week). This information is used to estimate weekly energy expenditure (kcal).

#### 2.3.6. Sleep

The Pittsburgh Sleep Quality Index (PSQI) is a widely used, reliable, and valid measure of sleep quality and disturbances over the previous month [27]. The PSQI also contains the following two items, which were used to calculate TIB: “During the past month, when have you usually gone to bed?” and “During the past month, when have you usually gotten up in the morning?” Previous research has documented a possible U-shaped association between sleep and obesity risk such that the greatest protection against obesity is achieving between seven and eight hours of nightly sleep [28]. Consistent with previous research, and to enable comparisons to the most protective sleep length (i.e., 7-8 hrs/night), TIB was categorized as follows: ≤6 hours/night, >6 to ≤7 hours/night, >7 to ≤8 hours/night, >8 to ≤9 hours/night, and >9 hours/night. 

### 2.4. Data Analysis

Analyses were conducted using PASW Statistics 18, Release 18.0.0 (SPSS, Inc., 2009, Chicago, IL, USA, http://www.spss.com/), and Stata/SE 12.0 for Windows (StataCorp, 2011, College Station, TX, USA, http://www.stata.com/). Both the PPAQ and the episodes of urinary incontinence variables were skewed. Thus for all analyses, log transformed values for each were used. Preliminary analyses examined the baseline association between demographic and lifestyle/health behavior variables with each of the outcomes (BMI, physical activity, and eating behaviors) using Pearson correlations or analyses of variance (ANOVA). Variables that were associated (*P* < 0.10) with at least one of the outcome variables were included in all subsequent analyses. The following factors were assessed given their potential association with BMI, physical activity, and/or eating behaviors: race, education, relationship status, employment, Beck Depression Inventory score, reported alcohol consumption, and frequency of snoring/coughing loudly (i.e., as a marker of sleep disordered breathing). Because all women had urinary incontinence, preliminary analyses were run for both total nightly and total weekly incontinence episodes with outcome variables. “Total weekly urinary incontinence episodes” were more strongly associated with outcome variables and were therefore retained (instead of total nightly episodes) for subsequent analyses. There was no association between incontinence type and any of the outcome variables so it was not retained in analyses. 

Separate regressions were constructed for each outcome variable. Based on our a priori hypothesis that the response would be U-shaped, we used a quadratic orthogonal contrast to assess the overall association of TIB with each outcome after controlling for possible confounding factors and tested for pairwise differences between TIB groups. Given that the PPAQ was log transformed to address right skewness, regression results were back transformed to obtain adjusted percent between-group differences. Statistical significance for regression models was set at *P* < 0.05.

## 3. Results

The 318 randomized women had mean (±SD) age of 53.0 (10.2), and 55% had no college education. Sixty-eight percent reported current alcohol consumption, and 30% reported coughing/snoring at least once per week. Mean Beck Depression Index was 7.5 (5.9). On average, participants reported sleeping 7.8 (1.3) hours per night, expending 1054 (791) kcal in physical activity per week and consuming approximately 2,080 (981) kilocalories per day. Diets consisted, on average, of 40% (8%) kcal from fat, 15% (3%) kcal from protein, and 45% (8%) kcal from carbohydrates. On average, 13% (9%) of calories were reported to be from sweets.  Table 1 shows unadjusted means for BMI, physical activity, and dietary variables by sleep duration category.

### 3.1. Association between TIB, Physical Activity, Dietary Intake, and BMI

Table 2 presents findings from adjusted analyses for both BMI and PPAQ scores. After adjustment for race, education, suspected sleep disordered breathing, age, total weekly urinary incontinence episodes, BDI score, and alcohol use, average BMI was 2.2 kg/m^2^ higher in the group reporting ≤6 hours TIB per night (*P* = 0.04) than in the referent category, and the average physical activity score was 55% lower (*P* = 0.05). TIB was not associated with any of the dietary composition variables measured on the Block FFQ. 

## 4. Discussion

In a sample of women with urinary incontinence presenting for weight loss treatment, reporting six hours or less TIB each night is associated with a higher BMI. This finding persisted after controlling for a number of possible confounding factors, including total weekly urinary incontinence episodes. The present findings are consistent with previous research, and when considered in light of experimental studies with normal weight adults [5, 13, 14], they highlight the importance of exploring whether insufficient sleep could enhance behavioral weight loss treatment.

Although several studies have demonstrated an increased risk of obesity with short sleep, less is known regarding how sleep affects physical activity and eating behaviors. The present findings demonstrate that reported TIB of six hours or less was associated with a 55% reduction in physical activity compared to sleeping approximately seven to eight hours per night. Although one experimental study and one cross-sectional study [16] did not find differences in energy expenditure based on sleep duration [5], the present finding is consistent with three other studies. In two experimental studies with healthy normal weight men, partial sleep restriction was associated with decreased resting and postprandial energy expenditure [12] as well as decreased physical activity in free living (but not laboratory) conditions [11]. Furthermore, in a cross-sectional study with middle-aged and elderly women who were normal weight or overweight, short sleep was associated with report of decreased physical activity [17]. The present finding builds upon previous work by documenting this association between TIB and physical activity in a weight loss treatment seeking sample of overweight and obese women and suggests that sleep duration may be an important factor to consider when asking women to enhance physical activity within a weight loss program.

There was no association between shortened sleep length and dietary composition in the present study. This may be due to reliance on a self-report measure of food intake. However, similar to physical activity, previous findings [10, 11, 13, 14, 29] have been inconsistent, even when food intake has been observed and measured within controlled experimental settings. For example, while two experimental studies found that partial sleep restriction was associated with increased ad libitum intake [5, 14], two additional experimental studies found no dietary differences between partial sleep restriction and a rested condition [11, 12]. Taken together, mixed findings to date suggest that the association between sleep and eating behaviors in adults may be small or variable, and the association between sleep and BMI may not be solely attributable to eating pathways. Additional studies that include more comprehensive, objective measures of food intake are clearly needed.

Although the present study has several strengths, including its focus on a treatment-seeking population and extension of previous research by assessing not only BMI but also physical activity and dietary intake, there are several limitations. First, it focused on a sample of overweight and obese women with urinary incontinence. Although this represents a select population, the consistency of findings with previous research, high prevalence of urinary incontinence in the general population, particularly in overweight women [30, 31], and predominance of women presenting for weight loss treatment suggest that findings may generalize to a large segment of the treatment-seeking population. Furthermore, although total weekly episodes of urinary incontinence were associated with BMI, it was not associated with sleep duration, and findings regarding the association between sleep and BMI persisted even after controlling for urinary incontinence. Second, measures of sleep, physical activity, and eating behaviors were based on self-report, which may be subject to reporter bias. Third, the cross-sectional nature of the study cannot demonstrate a causal link between sleep and either physical activity or obesity status. Prospective, randomized trials that manipulate sleep length in adults are needed prior to determining that enhancing sleep can assist with weight loss. 

In conclusion, among women presenting for behavioral weight loss treatment, self-report of shorter TIB (i.e., 6 hours or less each night) was associated with higher BMI and lower physical activity. Although there are limited data on enhancing sleep duration as a component of behavioral weight loss treatment, mounting evidence suggests that increasing sleep duration as an adjunct approach for improving weight regulation and physical activity warrants further study.

## Figures and Tables

**Table 1:** Unadjusted mean (standard deviations) for BMI, physical activity, and dietary variables by sleep categories (*N* = 318).

	Sleep duration categories					
	≤6 hrs (*n* = 35)	>6 to ≤7 hrs (*n* = 66)	>7 to ≤8 hrs (*n* = 111)	>8 to ≤9 hrs (*n* = 70)	>9 hrs (*n* = 36)	*P* value^a^
Body mass index, mean (SD)	38.8 (5.5)	36.0 (5.0)	36.1 (5.8)	35.8 (5.6)	36.3 (5.8)	0.04
Paffenbarger physical activity (kcal burned), mean (SD)	556.0 (889.7)	752.9 (1148.4)	934.6 (1215.3)	713.7 (746.4)	788.3 (981.4)	0.07
block FFQ, mean (SD)						
kcal	2064.8 (961.0)	1917.5 (903.2)	2219.2 (1055.0)	1965.4 (883.3)	2187.0 (1054.3)	NS
% kcal from fat	41.4 (8.2)	39.8 (7.0)	40.2 (7.1)	41.1 (8.37)	41.3 (7.9)	NS
% kcal from protein	15.0 (2.8)	15.6 (3.3)	15.7 (3.0)	15.5 (3.5)	15.4 (3.0)	NS
% kcal from carbohydrates	45.4 (8.1)	45.8 (7.5)	44.9 (7.4)	43.9 (9.3)	43.8 (8.9)	NS
% kcal from sweets	14.4 (7.8)	12.9 (9.0)	12.8 (8.9)	12.0 (8.8)	13.6 (12.9)	NS

^a^Statistical significance was determined using a quadratic orthogonal contrast, motivated by a priori hypothesis of a U-shaped response.

**Table 2:** Differences in average BMI and Paffenbarger physical activity score between sleep duration groups (*N* = 318).

Sleep duration groups	BMI			Paffenbarger PA		
	Difference (kg/m^2^)^a^	95% CI	*P* value	Percent reduction^b^	95% CI	*P* value
≤6 h/night	2.2	0.1–4.3	0.04	54.6	0.0, 79.4	0.05
>6 to ≤7 h/night	−0.2	−1.8, 1.4	NS	40.4	−11.1, 68.0	0.10
>7 to ≤8 h/night	ref	ref	ref	ref	ref	ref
>8 to ≤9 h/night	0.1	−1.5, 1.7	NS	34.7	−21.0, 64.8	NS
>9 h/night	0.6	−1.4, 2.6	NS	45.8	−17.7, 75.0	NS

*Note*. Findings adjust for race, education, suspected apnea, age, urinary incontinence, BDI score, and alcohol use.

^a^Difference in BMI. ^b^Back transformed.
